# A model to predict the incidence of allergic rhinitis based on meteorological factors

**DOI:** 10.1038/s41598-017-10721-3

**Published:** 2017-08-30

**Authors:** Yuhui Ouyang, Jin Li, Deshan Zhang, Erzhong Fan, Ying Li, Luo Zhang

**Affiliations:** 1 Department of Otolaryngology Head and Neck Surgery and department of Allergy, Beijing TongRen Hospital, Affiliated to the Capital University of Medical Science, Beijing, 100730 China; 2 Beijing Key Laboratory of Nasal Diseases, Beijing Institute of Otolaryngology, Beijing, 100005 China; 3 Beijing Weather Information Service, Beijing, 100089 China

## Abstract

Meteorological factors have been shown to affect the physiology, distribution, and amounts of inhaled allergens. The aim of this study was to develop a model to predict the trends for onset of allergic rhinitis (AR) patients. A total of 10,914 consecutive AR outpatients were assessed for the number of daily patient visits over a period of 4 years. Meteorological data were used to assess the relationship between meteorological factors and AR incidence by time-series data and regression analysis. Predictive models for incidence of AR were established in pollen-, dust mite- and mould-sensitive groups of patients, and the predictive performances of meteorological factors on the incidence of AR were estimated using root mean squared errors (RMSEs). The incidence of pollen-, dust mites- and mould-sensitive AR patients was significantly correlated with minimum temperature, vapour pressure, and sea-level pressure, respectively. The correlation between comprehensive meteorological parametric (CMP) and incidence of AR was higher than the correlation between the individual meteorological parameters and AR incidence. CMP had higher performance than individual meteorological parameters for predicting the incidence of AR patients. These findings suggest that the incidence of pollen-, dust mites- and mould-sensitive AR can be predicted employing models based on prevailing meteorological conditions.

## Introduction

Allergic rhinitis (AR) is an inflammatory disease of the nasal mucosa, induced by an immunoglobulin E (IgE)-mediated reaction in allergen-sensitized subjects^1^. AR is characterized by sneezing, rhinorrhea, nasal congestion and nasal pruritus, which are often accompanied by ocular pruritus, redness and/or lacrimation in 60–70% of patients. Prevalence of AR has increased over the last few decades, with about 25% of the worldwide population affected^2, 3^, including in China^1^. AR significantly impacts the quality of life of affected individuals, affecting work and social life, causing multiple comorbidities, and requiring considerable healthcare resources^4–6^. Several studies have demonstrated that inhaled allergens, including pollen, mould, and dust mites, as well as exposure to cockroach and animal wool in the environment, may trigger the exacerbation of AR attacks^7–9^. The main inhaled allergens of AR in Beijing are from pollen, dust mite and mold^3^. Other studies have demonstratedmeteorological factors such as temperature, irradiance, and relative humidity toaffect the physiology, distribution, and amounts of these allergens^1, 10–12^, suggesting that meteorological factors are associated with allergic disease outbreaks and severity. Hjelmroos and colleagues^12^ demonstrated that birch trees growing at higher temperatures produced pollen with an increased content of major allergen, which also had higher allergenicity. Furthermore, for pollen allergens, the positioning of the branches also influenced the antigenic proteins and allergens produced; with the greatest amounts of antigenic proteins and allergens being extracted from pollen present in the south-facing branches and allergens decreasing progressively in pollen from west- through east- to north-facing branches^12^. Similarly, several studies have reported that heavy rainfalls and thunderstorms are associated with increased number and severity of asthma attacks, both in adults and children^13–16^. It has been suggested that under wet conditions or during thunderstorms, pollen grains may rupture by osmotic shock and release into the atmosphere respirable, allergen-carrying cytoplasmic starch granules (0.5–2.5 µm) or other paucimicronic components that can reach the lower airways and induce asthma reactions in pollinosispatients^14^. However, appropriate models for predictingincidence of AR are scarce, and urgently needed especially in China with its increasing population of AR patients.

The aim of this study was thus to develop a model for predicting the trends for onset of AR patients, based on correlation between meteorological factors and incidence of AR in patients sensitized to pollen, mould and house dust mite allergens.

## Materials and Methods

### Clinical data

#### Study population

The study population comprised 10,914 consecutive outpatients (aged 6 to 60 years) recruited from the Allergic Rhinitis Treatment Centre of Beijing TongRen Hospital, during the period from February 4, 2007 to November 22, 2010. The patients who had clinical symptoms of AR underwent SPT for a diagnosis of AR. All patients with a confirmed diagnosis of AR, based on medical history and positive skin prick tests (SPTs) to specified allergens^17^, were subsequently recruited to the study on the same day. Repeating patients were identified according to the department’s computerised patient database and were excluded. The vast majority of the patients (96.9%; 10576/10914) were residents of Beijing, with the remaining subjects (3.1%; 338/10914) from other cities. These patients were included in the study, because they had resided in Beijing in the recent three years, when they were recruited into the study.

To be included into the study, all patients had to demonstrate clinical symptoms of AR, such as sneezing, itchy and runny nose, and nasal congestion; and positive SPT results to relevant allergens. The patients were also required to complete a standardized questionnaire (including details of demographic characteristics, residency, recent outbreaks time, family history of allergic diseases, symptoms of rhinitis and medical history) under the guidance of doctors and not have taken any drug, which might affect SPT results (e.g., antihistamines and corticosteroids), for at least one week prior to SPT. The onset of AR was determined according to the questionnaire. Fifty seven AR subjects had failed to fill in the questionnaire appropriately and were therefore excluded from the study.

The study protocol was approved by the Ethics Committee of Beijing TongRen Hospital and performed in accordance with the guidelines of the World Medical Association’s Declaration of Helsinki. All patients gave written informed consent prior to inclusion in the study and all patient information was anonymized prior to analysis.

#### SPT and grouping

SPT was performed according to standardized procedures on the day of visit for diagnosis of allergy, using 21 inhalant allergens (Merck, Darmstadt, Germany). Histamine diphosphate (1 mg/mL) and PBS (1 mg/mL) were used as positive and negative controls, respectively. According to international guidelines, positive reaction was defined as a mean wheal diameter exceeding by 3 mm or more than the negative control value. Reactions were graded from +1 to +3 level (+1, erythema ≤20 mm diameter; +2, erythema >20 mm diameter; +3, wheal and erythema) according to Stytis *et al*.^18^. Skin tests were graded after 20 minutes in comparison to the positive control’s wheal diameter.

AR patients were divided into 5 groups based on their SPT results for the most predominant sensitizing allergens; namely (i) pollen (including mixed trees, Mugwort, Humulus scandens, Ambrosia, Dandelion, Chenopodium album and mixed Gramineae), (ii) dust mite, (iii) mold (including Penicillium notatum, Aspergillus fumigates, Alternaria alternate, Curvularia lunata and Fusarium moniliforme), (iv) cockroach, and (v) animal wool allergen groups. For the purpose of this study we have primarily investigated only the first three groups because these allergens are the main causes of AR in Beijing and show some degree of regularity in their annual peaks^1^.

### Meteorological and pollen data

Meteorological data included daily maximum, average and minimum temperatures, relative humidity, vapour pressure, precipitation wind speed, sea-level pressure, and dew point temperature. The data were recorded daily at the meteorological observatory of Dongcheng District of Beijing sited in the centre of the city and reported by the Beijing Meteorological Administration. Daily mean values were used in the analyses^19^.

Similarly, data for pollen were collected from a monitoring station situated in the vicinity of the meteorological monitoring station. The pollen stations are managed by Beijing Meteorological Administration. Pollen grains were collected daily using a Durham sampler (Yamato Co. Ltd., Japan) and then counted under a microscope by reading the slides. Mean daily counts of tree pollen were used for analysis.

### Data analysis

**Figure 1:**
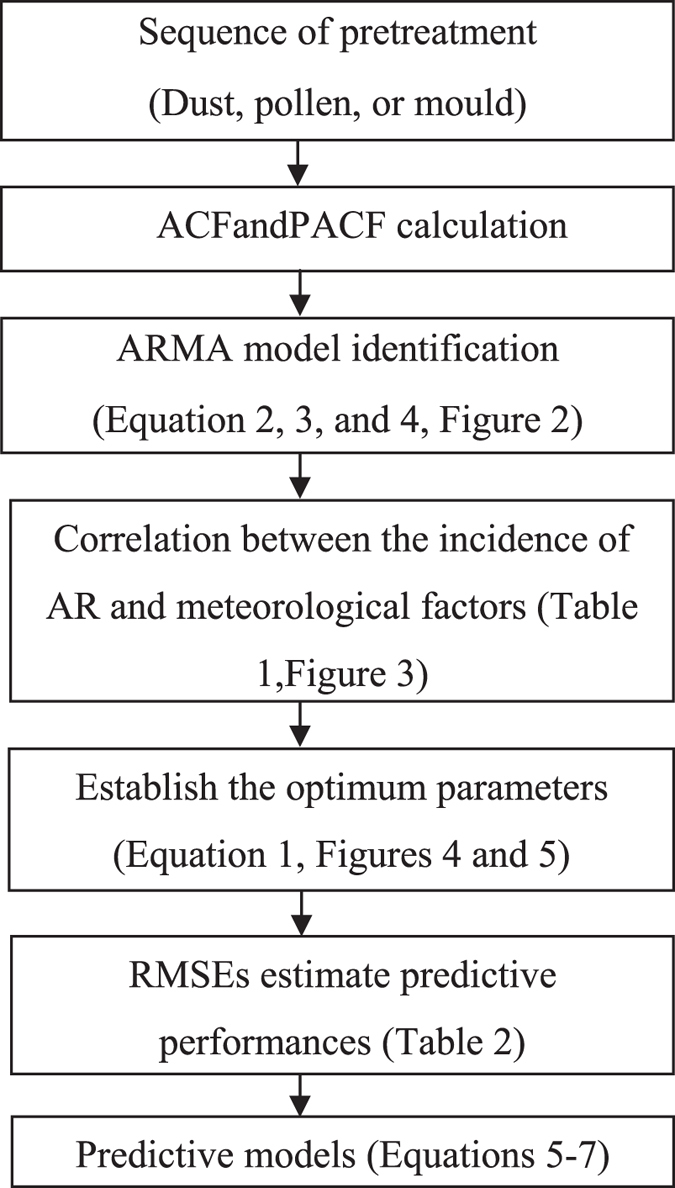
Design for development of models to predict the incidence of allergic rhinitis based on meteorological factors. ACF: Autocorrelation function; PACF: Partial autocorrelation function; ARMA: Autoregressive moving average; RMSEs: Root mean squared errors.

AR incidence was assessed as the number of different individual AR patients visiting our hospital from 2007 to 2010. In this regard data on “AR incidence”, were collected 92 times from 2007 to 2010 (once every two weeks) and were used in further analyses to develop the model predicting the incidence of allergic rhinitis based on meteorological factors, as shown in Fig. 1.

AR incidence was first assessed over time, using autocorrelation function (ACF) and partial autocorrelation function (PACF) combined with Bayesian information criteria (BIC) to determine the stationarity of data and fit model’s order. Autoregressive moving average (ARMA) model was considered to estimate the temporal trend of the data. White noise test was performed on the residuals to demonstrate the significance for the fitting model.

Correlation between incidence of AR symptoms and meteorological factors was analyzed and P values < 0.05 were considered to be significant. Comprehensive meteorological parametric (CMP) equations were established to summarize the complex collective effects of all prevailing meteorological factors on AR incidence (Equation 1),

1 $$CM{P}_{i}=\frac{{E}_{i}T{D}_{i}}{{P}_{i}}$$

where “Ei” represents the i^th^ average vapour pressure, “TDi” represents the i^th^ average minimum temperature, “Pi” represents the i^th^ average sea-level pressure, and “i” various recording times.

The predictive performances of meteorological factors and the CMP on the incidence of AR were estimated by root mean squared errors (RMSEs). The predictive models were built on about 70% of all samples randomly selected from the whole dataset, and were then validated on the rest 30% samples. The AR cases were divided into 4 grades according tothe probability distribution of AR (data not shown). The classification criteria were defined as follows: level I: AR cases ≤ 16; level II: AR cases = 17–52; level III: AR cases = 53–104; level IV: AR cases ≥ 105. The predictions of AR incidence were performed according to daily meteorological factors and the diagnostic values of the models. Statistical analyses were carried out using SAS 9.2 (SAS Institute Inc., USA) and OriginPro 9.0 (OriginLab, USA) and P < 0.05 was considered to be statistically significant.

## Results

### AR incidence over the course of 4 years in pollen, dust mite, and mould-sensitized patients

**Figure 2:**
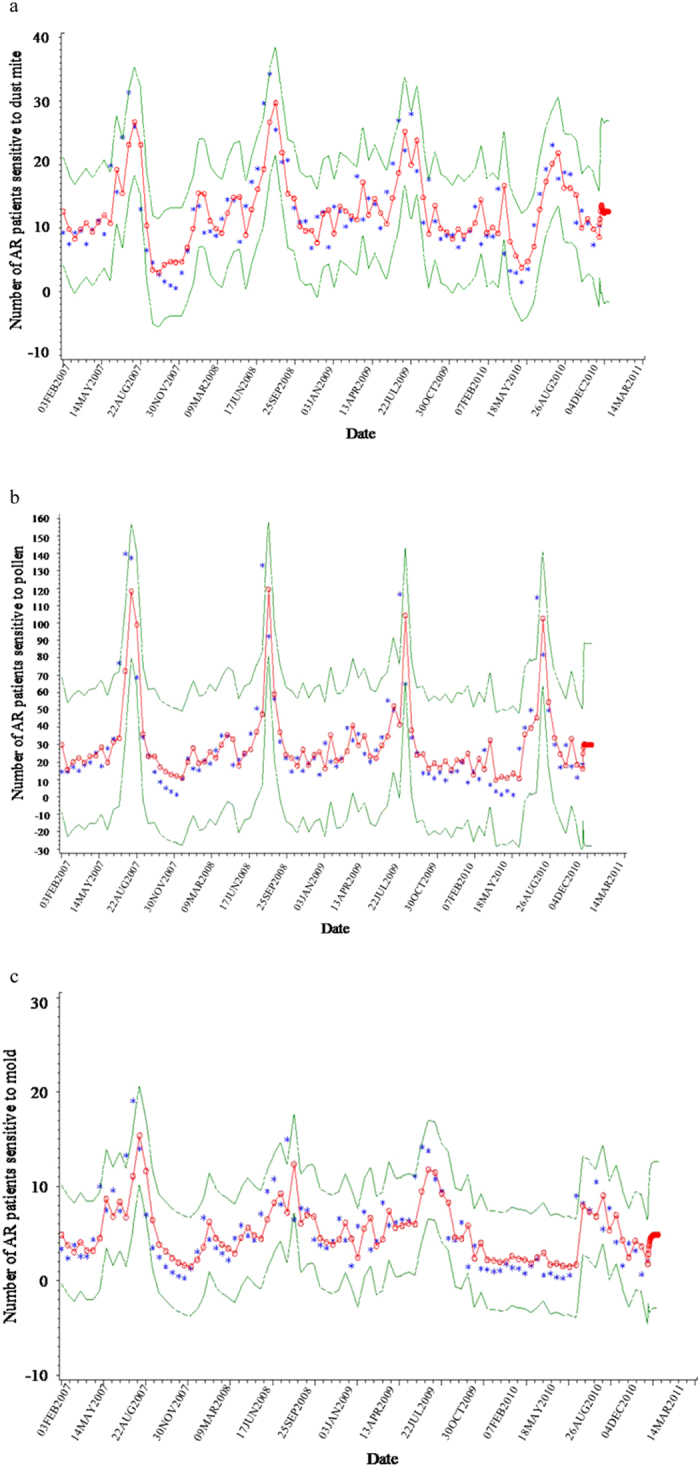
Fitting curves for incidence of AR induced by Dust mite (**a**), pollen (**b**), and mould (**c**). Blue dots, original data dots/points; red line, fitting curves of equation; green line, 95% CI of the fitting equation.

The demographic characteristics of the participants are shown in Table 1. The final analysis included 10914 screened patients. The mean age of the study subjects was 37.2 (±14.1 SD) years. There were 4,944 (45.3%) men and 5,970 women (54.7%). Of the test subjects, 3,547 (32.5%) patients had pollen allergy, 4,976 (45.6%) patients had dust mite allergy, and 2,391 (21.9) patients had mould allergy. The mean age of pollen, dust mite, and mould-sensitized patients was 39.3 (±13.7 SD), 33.5 (±11.6 SD), and 37.6 (±15.2 SD) years, respectively. AR incidence rates in patients sensitive to various allergens during the course of the study are shown in Fig. 2. AR incidence in patients sensitive to pollen, dust mite, and mould allergens increased from August to September, and peaked between August 20 and September 4; during which period Artemisia and Chenopodiaceae are the main pollen grains present in Beijing, China. Moreover, a smaller peak of AR incidence was also observed from May 5 to 19 for patients sensitized to pollen allergens, such as birch, Cypress and Salicaceae.

**Table 1:** Demographic Characteristics of patients.

	Total	Patients with main allergy		
		Pollen	Dust mite	Mould
N	10914	3547	4976	2391
Age (years)				
Mean	37.2	39.3	33.5	37.6
SD	14.1	13.7	11.6	15.2
Sex, men				
n	4944	1901	2234	1035
%	45.3	53.6	44.9	43.3

AR incidence in patients sensitive to pollen, dust mite, and mould allergens were assessed over time, using the respective regression equations as shown below:

2 $${\rm{AR}}\,{\rm{sensitive}}\,{\rm{to}}\,{\rm{pollen}}:{\rm{xt}}=0.83473{{\rm{\chi }}}_{t-1}\,\mbox{--}\,0.21551{{\rm{\chi }}}_{t-4}+{\varepsilon }_{t}$$

3 $${\rm{AR}}\,{\rm{sensitive}}\,{\rm{to}}\,{\rm{dust}}\,{\rm{mite}}:{\rm{xt}}=0.92557{{\rm{\chi }}}_{{\rm{t}}-{\rm{1}}}\,\mbox{--}\,0.27506{{\rm{\chi }}}_{{\rm{t}}-{\rm{2}}}+{\varepsilon }_{t}$$

4 $${\rm{AR}}\,{\rm{sensitive}}\,{\rm{to}}\,{\rm{mould}}:{\rm{xt}}=0.73918{{\rm{\chi }}}_{{\rm{t}}-{\rm{1}}}+{\varepsilon }_{t}$$

Where *ε*_*t*_ is white noise and xt is predicted trend of AR incidence; t (time unit) is bi-weekly.

**Figure 3:**
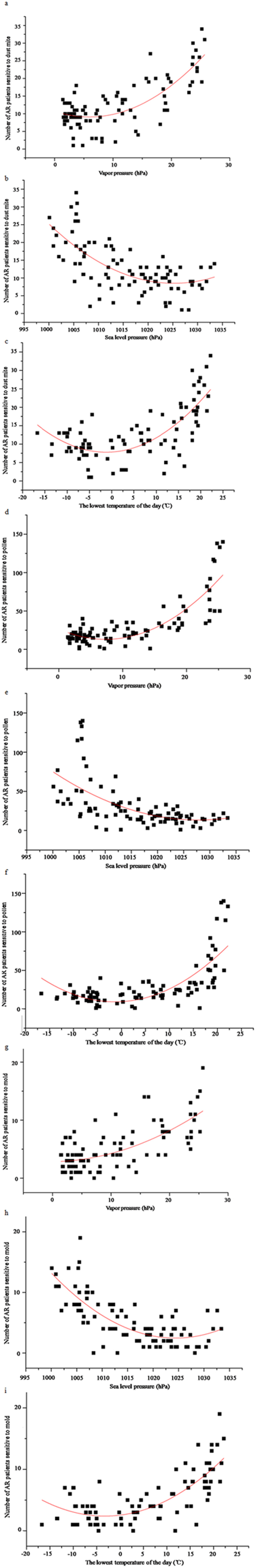
Correlation between AR incidence and meteorological factors. Dust mite (**a–c**), pollen (**d–f**) and mould (**g–i**) were assessed against vapour pressure (**a**,**d**,**g**), sea level pressure (**b**,**e**,**h**), and minimum daily temperature (**c**,**f**,**i**).

Figure 3 shows the model fitted curves for incidence of AR according to the type of allergen over the course of 4 years; with the green lines showing the confidence bands of the fitting equation.

### Correlation between AR incidence and meteorological parameters

Correlation between AR incidence and meteorological factors are presented in Table 2 and Fig. 3. The results showed that AR incidence in patients sensitive to pollen, dust mite, and mould allergens was significantly correlated with minimum temperature, vapour pressure, and sea-level pressure (P < 0.05). Sea-level pressure and AR incidence showed an early negative correlation; indicating that the lower the sea-level pressure values, the higher was the AR incidence. In contrast, minimum temperature, vapour pressure, and AR incidence presented a positive correlation; indicating that the higher the temperature or vapour pressure values, the higher was the AR incidence.

**Table 2:** Correlation between the incidence of AR symptoms and meteorological parameters.

Allergens	Minimum temperatures		Sea level pressure		Average vapour pressure		Comprehensive parameters	
	R-square	*F-* value	R-square	*F-* value	R-square	*F-* value	R-square	*F-* value
Pollen	0.576	60.4	0.315	41.3	0.700	103.8	0.750	133.6
Dust mite	0.551	54.5	0.368	52.4	0.598	66.3	0.600	66.8
Mould	0.565	57.7	0.450	73.6	0.477	40.6	0.539*	52.1

Quadratic curves were fitted on the number of AR patients against the meteorological parameters respectively (P < 0.001 for all quadratic models).

**Figure 4:**
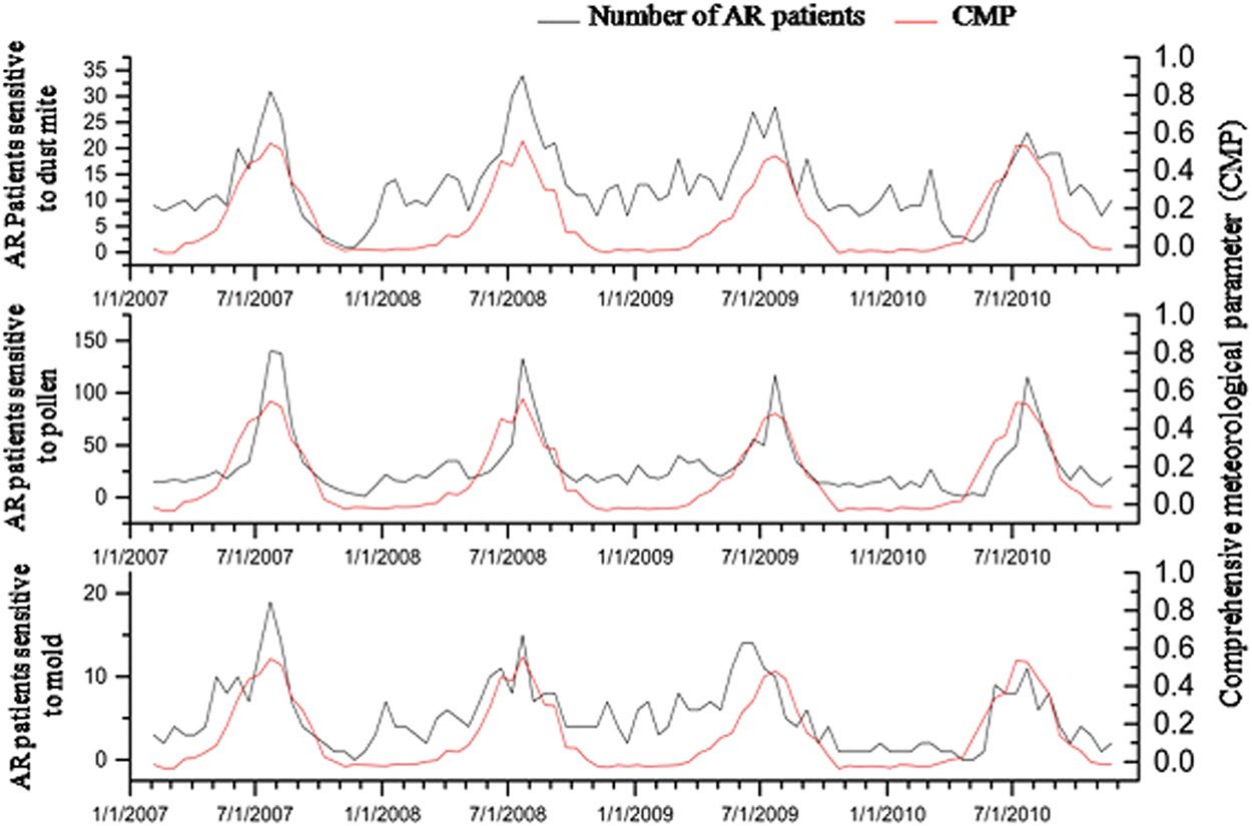
AR incidence in patients sensitive to dust mite, pollen and mould, over a period of 4 years. Red and black lines represent comprehensive meteorological parameters (CMP) and AR incidence, respectively.

**Figure 5:**
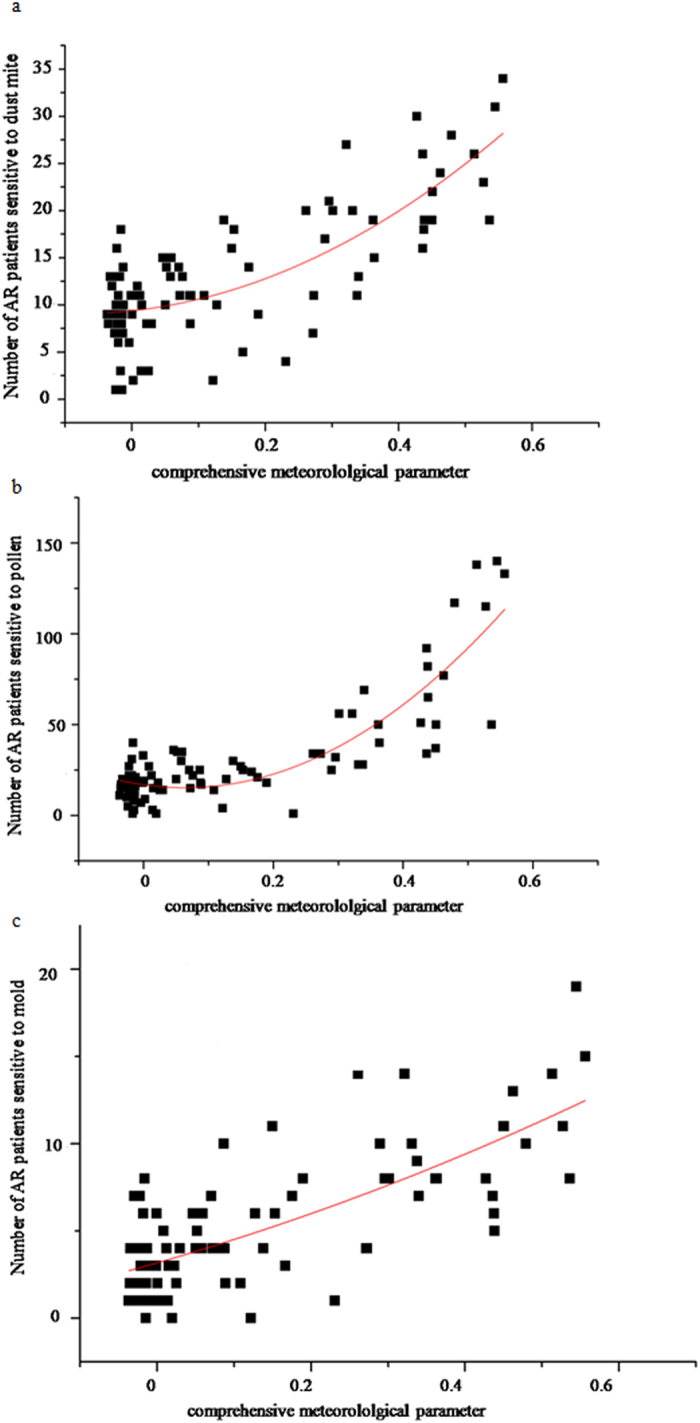
Correlation between CMP and AR incidence in dust mite- (**a**), pollen- (**b**), and mould-sensitized (**c**) patients.

CMP is a comprehensive parameter of above-mentioned meteorological factors. AR incidence in patients sensitive to pollen, dust mite, and mould allergens were significantly correlated with CMP (P < 0.05), and the correlation between CMP and incidence of AR was higher than the correlation between the individual meteorological parameters and AR incidence for the same period (Table 2, Figs 4 and 5).

When evaluating the predictive performance of individual meteorological factors and CMP, 67 samples (72.8%) were selected from the whole dataset randomly. The remaining 25 samples were used to validate the predictive models built on the 67 samples. When predicting the number of patients sensitive to pollen and mould, CMP had the highest performance (RMSEs: 12.78 and 2.57, respectively), while having the second highest performance (RMSE: 4.25) for predicting the number of patients sensitive to dust mites (Table 3). These results suggest that the AR incidence can be predicted. Furthermore, predictive models for incidence of AR in pollen, dust mite and mould groups were established as shown below:

5 $${{\rm{y}}}_{1}=404.45{{\rm{x}}}^{2}-51.32{\rm{x}}+16.88$$

6 $${{\rm{y}}}_{2}=47.20{{\rm{x}}}^{2}+7.59{\rm{x}}+9.36$$

7 $${{\rm{y}}}_{3}=7.35{{\rm{x}}}^{2}+12.64{\rm{x}}+3.17$$

where y_1_, y_2_, y_3_ are predicted values for AR incidence in pollen-, dust mite- and mould-sensitized groups, respectively; and “x” represents comprehensive meteorological parameters.

**Table 3:** Predictive performance (root mean squared error, RMSE) of meteorological parameters on the incidence of AR.

Allergens	Minimum temperatures	Sea level pressure	Average vapour pressure	Comprehensive parameters
Pollen	19.00	25.90	15.24	12.78
Dust mite	4.64	5.69	4.24	4.25
Mould	2.61	2.96	2.82	2.57

## Discussion and Conclusions

Our study first provided a prediction of the incidence of AR based on meteorological factors in Beijing. We assessed the associations between meteorological factors and AR incidence over a period of four years, in mostly residents from Beijing (96.9%) and to a smaller extent from neighbouring cities (3.1%). AR patients sensitized to pollen, dust mite and mould were investigated primarily because these allergens are the main causes of AR in Beijing ^3^and because they all show onset peaks between August and September (Fig. 2), which suggests that the incidence of AR may possibly be influenced by meteorological conditions. Indeed, model fitting analysis showed a high degree of fit for these allergens (Fig. 3), indicatingstationarity of the data over time, as well as the fitting model’s order. Then, based on the observed correlations between meteorological factors and incidence of AR, we set up a model for prediction of the incidence of AR.

Exposure to allergens may trigger the exacerbation of AR attacks; with increased concentrations of inhaled allergens being responsible for increased incidence and severity of allergic diseases^1, 20, 21^. In the current study, Beijing’s pollen monitoring data showed that air pollen concentrations peaked during the periods from August to September and from April to May. August and September constitute the blooming period for weeds, such as Artemisia and Humulus, whereas April and May is important for woody plants, such as birch, Cypress and Salicaceae^3^. However, as Beijing has a temperate climate, with the environment having higher humidity and temperature in August and September, these conditions are also suitable for dust mite and mould growth. Thus, the peaks of AR incidence noted in August to September and in May in the current study are consistent with the peaks of “allergenic protein” noted during these periods in the year.

Consistent with previous findings, our study has also demonstrated that the incidence of AR patients in the three groups significantly correlate with meteorological factors, minimum temperature, vapour pressure and sea-level pressure (Table 1). Indeed, we found that higher minimum temperature or vapour pressure values and smaller sea level pressure values were associated with higher incidence of AR. Furthermore, we estimated the optimum parameter - comprehensive meteorological parameter (CMP), andthe results showed correlation values between CMP and pollen, dust mite, mould allergens are higher than the correlation between the individual meteorological parameters and AR incidence for the same period (Table 1, Figs 4 and 5); the predictive performance of CMP showed higher performance than individual meteorological factors (RMSEs: 12.78, 2.57 and 4.25, respectively, Table 2). These results indicated that the incidence of AR sensitive to pollen, dust mite, mould allergens can be predicted based on meteorological factors CMP.

The finding from the present study that AR incidence peaks in the three groups of patients were well synchronized with the CMP peak, suggests that changes in meteorological conditions can result in plant, mould, and dust mite growth, and increased allergen release^22^, potentially affecting AR incidence. Climate change is a constant and ongoing process postulated to be mainly driven by human activities^23^. It was recently shown that climate change increases the prevalence of certain ailments, including respiratory diseases and allergic responses, affecting the most vulnerable populations i.e. the elderly, children, and less socioeconomically fortunate^24^. Although the potential of meteorological factors as predictors for incidence of AR symptoms may be useful in the development of correlation prediction models, meteorological parameters are only one area of influencing factors and also help raise awareness for the role of other factors such as extreme environmental pollution and possibly other less obvious factors on human health, especially AR. This is particularly so for air pollution (industrial pollution and vehicle emissions of gas pollutants and particulate matter), that is especially severe during May and the September - October period when anticyclonic weather situations occur most frequently over Beijing area, and substantially contribute to the higher occurrence of AR cases. However, this study is somewhat limited in that it is a single centre study with a relatively small cohort, and all meteorological parameters were assessed locally. The study is also somewhat limited in that it does not account for the effects of meteorological parameters on air pollution and the incidence of AR or the impact of the meteorological parameters on incidence of AR in the very young, elderly and socioeconomically disadvantaged subjects. Thus, further large multicentre studies assessing the various meteorological factors in several locations, and taking into account the presence of particularly air pollution levels at the time of monitoring, as well as the age and socioeconomic status of the subjects are needed to substantiate this model in the future.

In the present study, pollen was measured using a Durham sampler, which collects airborne pollen by a gravimetric method. Although, this technique is relatively simple and inexpensive, pollen sampling is influenced by wind speed, orientation of the sampler with respect to wind direction, as well as concentration of pollen in the air. In comparison, the Burkard sampler is comparatively new and collects airborne pollen by a volumetric method, which involves drawing ambient air into the sampler at 10 l/min. Although sampling efficiency is comparatively high, the wind speed and the particle size shape and density have been reported to affect sampling efficiency of the Burkhard sampler^25^. Despite the differences in the sampling techniques, longitudinal studies by Crispen and colleagues^26^ and Kishikawa and colleagues^27^, have demonstrated that both techniques were equally suitable and comparable in monitoring airborne pollen under local conditions. Indeed, the study by Crispen and colleagues^25^ compared local pollen counts in Missoula, Montana in 1978 determined using a Durham sampler with those determined in 2006 using a Burkard sampler and demonstrated that the two sampling methods were comparable when reporting relative frequency of occurrence. Furthermore, Kishikawa and colleagues^27^ indicated that Durham’s technique was not inferior to Burkard’s technique, but rather defined the start of pollen dispersion more distinctly.

In conclusion, to our knowledge this is the first model, which provides a sound methodological basis for the prediction of the incidence of AR based on meteorological factors in Beijing (China). In addition to meteorological factors, other parameters affecting the onset time of AR; for example environmental pollution, which is an extreme risk for respiratory diseases in megacities such as Beijing, and personal health conditions should be further assessed to confirm the feasibility of the prediction model described here.
